# Pancreatic Cancer Heterogeneity Can Be Explained Beyond the Genome

**DOI:** 10.3389/fonc.2019.00246

**Published:** 2019-04-05

**Authors:** Natalia Anahi Juiz, Juan Iovanna, Nelson Dusetti

**Affiliations:** Centre de Recherche en Cancérologie de Marseille (CRCM), INSERM U1068, CNRS UMR 7258, Institut Paoli-Calmettes, Parc Scientifique et Technologique de Luminy, Aix-Marseille Université, Marseille, France

**Keywords:** pancreatic cancer, epigenetics, tumor heterogeneity, therapy, biomarkers

## Abstract

Pancreatic ductal adenocarcinoma (PDAC) remains a major health problem because it induces almost systematic mortality. Carcinogenesis begins with genetic aberrations which trigger epigenetic modifications. While genetic mutations initiate tumorigenesis, they are unable to explain the vast heterogeneity observed among PDAC patients. Instead, epigenetic changes drive transcriptomic alterations that can regulate the malignant phenotype. The contribution of factors from the environment and tumor microenvironment defines different epigenetic landscapes that outline two clinical subtypes: basal, with the worst prognosis, and classical. The epigenetic nature of PDAC, as a reversible phenomenon, encouraged several studies to test epidrugs. However, these drugs lack specificity and although there are epigenetic patterns shared by all PDAC tumors, there are others that are specific to each subtype. Molecular characterization of the epigenetic mechanisms underlying PDAC heterogeneity could be an invaluable tool to predict personalized therapies, stratify patients and search for novel therapies with more specific phenotype-based targets. Novel therapeutic strategies using current anticancer compounds or existing drugs used in other pathologies, alone or in combination, could be used to kill tumor cells or convert aggressive tumors into a more benign phenotype.

## Introduction

Approximately 350,000 people worldwide die every year due to pancreatic ductal adenocarcinoma (PDAC), making it the most lethal cancer (1). Despite all the efforts made in research over the last few decades, its prognosis has not significantly improved, with a variable survival time after diagnosis ranging from 2 to 3 months to more than 5 years (only in 5% of cases). One of the principal problems facing this disease is the heterogeneity observed among patients regarding symptoms, clinical evolution, predisposition to early metastasis, and sensitivity to treatments. Heterogeneity can arise at multiple stages of tumor evolution, from the first genetic mutations that gave origin to the tumor, its interaction with the microenvironment and as a result of selection pressure and clonal expansion (2). Nevertheless, two clinical subtypes of PDAC have been characterized: basal, with the worst prognosis, and classical. Less than 10% of PDAC patients respond to gemcitabine. This percentage increases three times with FOLFIRINOX, a protocol that combines four different drugs (5-FU, leucovorin, irinotecan, and oxaliplatin) (3). Hence, more than 70% of patients do not respond to the current therapies and even worse, failure of the first line chemotherapy leads to an acceleration of tumor growth conducive to a resistant and metastatic tumor. At present, there are no methods that can predict a patient's response to treatment or their prognosis, therefore clinicians choose therapy protocols based only on the patient's general condition and stage of progression. Thus, methods to predict if a patient will respond to the current chemotherapies (and which one) must be urgently developed; and it is pertinent that a treatment is found for the majority of patients for whom these therapies do not work.

Uncovering the mechanisms underlying tumoral heterogeneity has become a hallmark in cancer research (4), and we are convinced that it would be a good starting point in the selection of personalized therapeutic strategies. New insights in this field suggest that the frequent genetic aberrations present in PDAC samples, especially those in genes related to the epigenetic machinery, trigger the first epigenetic changes. However, we think that these epigenetic modifications are the driving forces that alter gene expression and define the malignant PDAC phenotypes. In fact in a recent publication Nicolle et al. (5) present a deep analysis of pancreatic cancer xenografts showing that tumor subtypes are better defined by specific epigenetic, transcriptional, and stromal landscapes than by gene mutations. They reveal also interesting potential therapeutic targets in the cross-talk between tumor and stromal cells.

## The Epigenetic Landscape Underlying PDAC as a Target for Patient Treatment

Epigenetics is defined as all the changes in phenotype and gene expression which are not due to alterations in the DNA sequence (6). These changes occur by a variety of mechanisms, frequently involving an effect on gene expression patterns: DNA methylation, chromatin remodeling, histone modifications and non-coding RNA molecules (lncRNAs and miRNAs). A great advantage that can be taken from the fact that the epigenome is the main factor responsible for PDAC phenotypes is that it is a reversible phenomenon, whilst genetic mutations are not. Thus, the development of epidrugs to modify aberrant epigenetic states represents a real opportunity to overcome PDAC. Adding further support for this hypothesis, an integrative analysis using ChIP-seq to characterize histone modifications, DNA methylation profiling and RNA-seq (7) showed that, as the result of activated epigenetic states produced by driver mutations, the PDAC epigenome is characterized by an upregulation of several epigenetic regulators with a clear feedback among them: DNA methyltransferases (DNMTs), histone methyltransferases and acetyltransferases, and non-coding RNAs.

### Histone Modifications

Some of the epigenetic regulators found to be upregulated in PDAC are the H3K4 methyltransferases MLL2 and SETD3, and the H3K acetyltransferase KAT2A, all of which activate transcription (7). Another regulator over-expressed in all PDAC samples is enhancer of zeste homolog 2 (EZH2). This enzyme is the functional enzymatic component of the chromatin remodeling polycomb repressive complex 2 subunit (PRC2), and it catalyzes the trimethylation of H3K27. Targeting polycomb modifications with epidrugs is of particular interest, as polycomb-repressed complexes have been found to silence tumor suppressor genes and hedgehog pathway genes. A first-in-class oral selective EZH2 inhibitor, tazemetostat, has been tested in a phase 1 study. This drug showed favorable results with a good safety profile and antitumor activity in patients with refractory B-cell non-Hodgkin lymphoma and advanced solid tumors (8). However, at present there are no reports of its use in PDAC pathology. Another promising approach is the implementation of amphipathic helical peptides. For example, NUPR1 is a protein that is over-expressed during acute pancreatitis (9), it is implicated in chromatin remodeling via its interaction with polycomb-group proteins, especially the C-terminal region of ring finger protein 1 B (C-RING1B) (10). It has been proven that helical peptides designed to target the intrinsically disordered NUPR1 protein inhibited its interaction with C-RING1B (11). Other epidrugs targeting histone marks have also been tested. For example, treatment of PDAC cells with chaetocin, a pan-H3K9me inhibitor, reduced cell growth. Interestingly, when combined with MLN8237 (alisertib) which targets aurora kinase A (AURKA), a key enzyme that regulates normal mitotic progression, the cytotoxic effect increased (12).

Histone deacetylase 1 (HDAC1) is another epigenetic modifier that is over-expressed in PDAC samples and may deregulate the histone acetylation pattern (7). In particular, higher expression levels of HDAC 1, 7, or 8 are associated with worse overall survival (13). Inhibiting HDACs could result in the activation of tumor suppressor genes leading to an inhibition of tumor cell proliferation. Until now, pancreatic cancer research has mostly focused on acetylation marks by studying HDAC inhibitors and proteins containing the bromodomain and extraterminal domain (BET), which recognizes or “reads” acetylation marks (14). It has been shown that the BET inhibitor JQ1 (also known as TEN-010 or thienotriazolodiazepine) suppresses the tumor growth in patient-derived xenograft models (15). Moreover, a synergistic effect on cell death and suppression of advanced PDAC was observed when JQ1 was combined with SAHA, an HDAC inhibitor also known as vorinostat that has already been approved by the United States Food and Drug Administration (FDA) (16). A recent study also demonstrated that another drug that targets HDACs, trichostatin A (TSA), increased apoptosis in MIA PaCa-2 and PANC-1 cell lines with better efficacy than SAHA, and moreover it enhanced the sensitivity of PDACs cells to gemcitabine (13). As BET bromodomains are determinants of c-MYC transcription (17), it is not surprising that cells from PDAC patients with high levels of MYC expression were more sensitive to JQ1 compared to cells with lower levels of MYC expression (18). The remarkable differences in results obtained for each tumor sample when studying cohorts of patients reveals the importance of patient stratification to select an efficient chemotherapy, and the limitations of extrapolating results obtained from only a few cell lines.

### Non-coding RNAs

Non-coding RNA (ncRNA) transcripts play a role as epigenetic modifiers regulating gene expression by different mechanisms, including interacting with histone modifying complexes or with DNMTs (19). These molecules are of special interest because targeting them leads to important downstream consequences in gene expression, and they can also be exogenously restituted. Among ncRNAs, the best studied are the microRNAs (miRNAs) that act as posttranscriptional repressors. Several of them are known tumor-suppressors, and their downregulation is implicated in the initiation and progression of PDAC. For example, in precursor lesions of PDAC miR-148 is repressed by DNA hypermethylation (20), and alongside miR-217 and miR-375, its downregulation is a meta-signature of PDAC (21). Different delivery strategies can be performed to restitute miRNA expression levels. One is the use of “nanovectors” that consist of lipid nanoparticles. These have been successfully used to deliver miR-34a, from the p53 transcriptional network, and the miR-143/145 cluster, known to downregulate KRAS2 expression, into cancer cells (22). The restitution of these miRNAs increased apoptosis and decreased proliferation in MIA PaCa-2 subcutaneous xenografts. In addition, miR-145 can been restored in human pancreatic cancer cells using a magnetic nanoparticle formulation (23). Silencing upregulated miRNA-21 and miRNA-221 in PDAC cells lines with antisense oligonucleotides also led to a significant inhibition of primary tumor growth and metastasis in gemcitabine resistant cells (24). Although miRNA-based therapy has been tested in preclinical studies, at present no clinical trials have been performed to test its use against PDAC (25).

### DNA Methylation

The DNA methylation is an epigenetic mark that causes gene silencing by retaining DNA in a transcriptionally quiescent state. In tumoral cells transcription of one of the five members DNMTs, the DNMT1 is increased, and higher levels of this enzyme correlate to poor clinical outcome (7, 26). Transference of a methyl group to the nucleotide cytosine converts DNA into a methylated form that is inaccessible to transcription factors (TFs) and blocks transcription. In cancer, specific DNA methylation patterns are known to silence oncogenes and tumor-suppressors (27). Interestingly, both the potent DNMT1 inhibitor, azacitidine, and its deoxy derivative, decitabine, are FDA approved epigenetic modulators, and their efficacy in the treatment of myelodysplastic syndromes has been tested in a phase III clinical trial (28). In solid tumors such as colorectal, breast, and lung cancers results from clinical trials and preclinical studies putatively suggest that these epidrugs can sensitize tumors to current chemo and immunotherapies (29–31). In PDAC cells, epigenetic reprogramming, by DNMT1 knockdown and by administration of the demethylating agent 5-azacytidine (5-AZA), inhibits tumor growth and also sensitizes resistant cell lines to gemcitabine (32). Zebularine, a known DNA methylation inhibitor, has been shown to have anticancer effects on established PDAC cell lines (33), but these results could not be reproduced on primary low-passage PDAC cultures derived from low-passage patient-derived xenograft tumors (34). However, zebularine drives PDAC stem cells to a more proliferative phenotype with increased sensitivity to current chemotherapies. A recent study (35) also showed an improvement in the response of MIA PaCa-2 and PANC-1 cell lines to irinotecan after sensitization with guadecitabine (SGI-110), a next generation epigenetic modulator with a longer half-life and better tolerability than 5-AZA (36). Nevertheless, data obtained from modifying DNA methylation patterns in cell lines may not reflect the heterogeneity present among patients and so, its extrapolation to clinics is uncertain. A study by Gayet et al. (37) aimed to identify an expression profile that could serve as a treatment response predictor in PDAC. They found a subgroup of PDAC tumors that were sensitive to the well-studied decitabine while other tumors were not. Interestingly, the sensitive tumors showed no correlation with DNMT1, DNMT3A, or DNMT3B expression, but they presented a specific transcriptomic profile.

The development of epidrugs faces several challenges, one of the most important being the lack of specificity: all epigenetic therapies affect the total genome (38). A deeper understanding of these epigenetic mechanisms could bring new and more specific therapeutic targets. Furthermore, heterogeneity should be taken into account because it is unlikely that one epidrug, alone or in combination with current therapies, will be efficient for all tumors. In cohort studies targeting epigenetic marks important differences were observed in the patients' response to treatments (18, 37). These results highlight the importance of implementing molecular characterization to identify biomarkers and classify tumors.

## Classifying Tumors: The First Step Toward Personalized Treatment

Part of the heterogeneity observed among PDAC patients can be explained by the classification into two different subgroups based on clinical outcome and therapeutic responses, named basal and classical subtypes. Patients with the basal subtype have a worse prognosis, but they respond better to adjuvant therapy compared to patients with the classical subtype. We know now that these PDAC phenotypes are defined by distinct epigenetic landscapes, and particularly by DNA methylation patterns, which are transduced at the transcriptional level and alter the interaction between the tumor and its stroma (5). However, the first attempts to stratify PDAC tumors were based on genetic mutations. Waddell et al. (39) classified samples in four subtypes (stable, locally rearranged, scattered, and unstable) with potential clinical utility. They defined another group called “on-genotype,” composed by unstable and/or high BRCA mutational signature genomes associated with response to platinum-based therapy. Nevertheless, other exome sequencing studies were carried out that confirmed relatively conserved mutated genes in PDAC (KRAS, TP53, SMAD4, ARID1A, and CDKN2A) (40, 41), but no clinically valuable tumor classification has emerged from these thousands of mutations and rearrangements. In addition, other genomic properties such as the chromosomal instability index and copy number aberrations show no association with any PDAC subtype (5). The lack of genetic support for the major clinical phenotypes of PDAC means that the search for the origin of PDAC heterogeneity must focus on other mechanisms regulated at a post-genetic level.

### The Epigenetic Landscape Can Define PDAC Phenotypes

**Figure 1:**
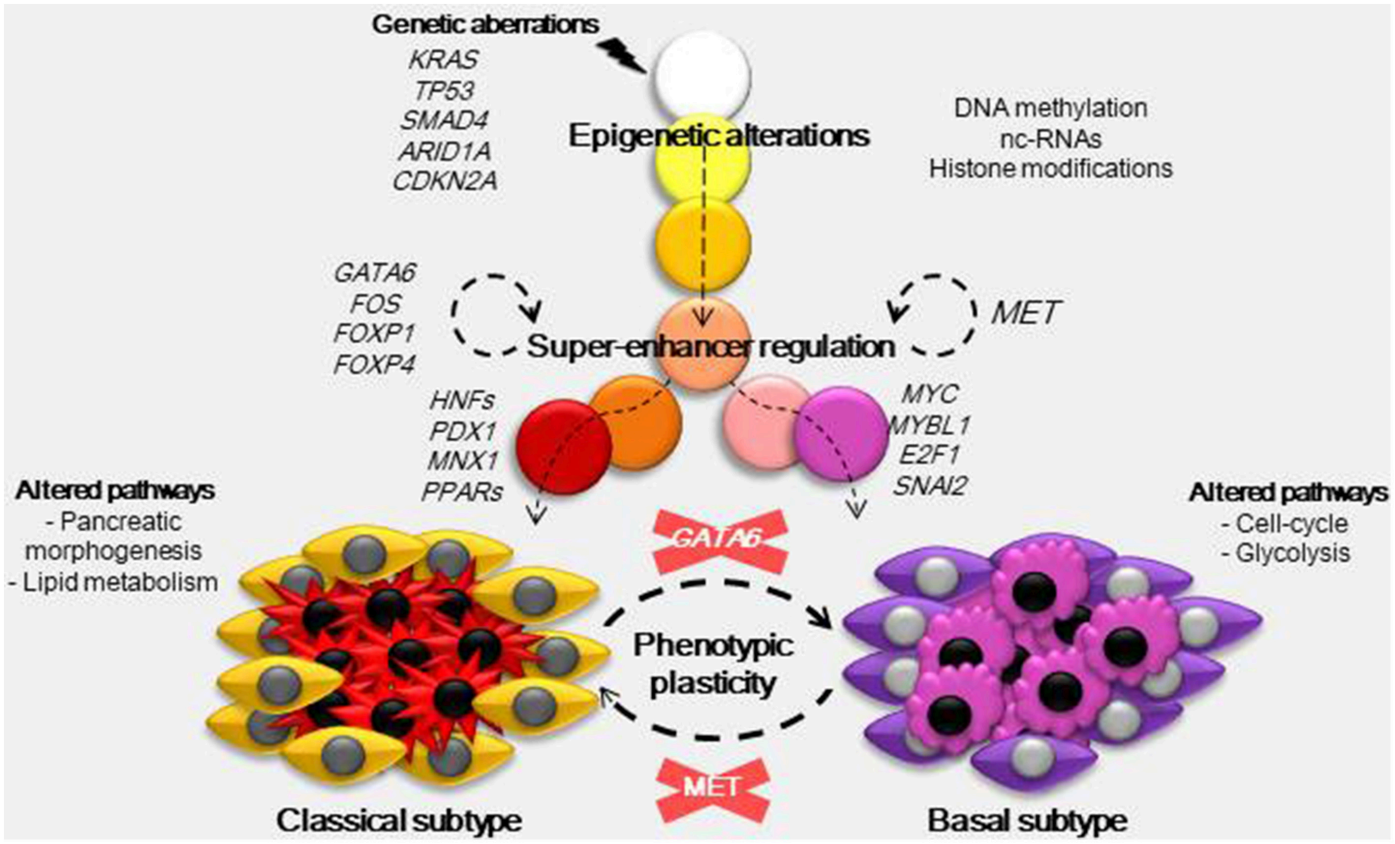
Developmental model for PDAC phenotypes. Carcinogenesis is initiated by a combination of certain mutations, frequently in genes such as KRAS, TP53, SMAD4, ARID1A, and CDKN2A. These mutations trigger epigenetic alterations by different mechanisms (DNA methylation, nc-RNAs, and histone modifications) and, combined with factors from the environment and tumor microenvironment, they establish subtype-specific epigenetic landscapes. These epigenetic landscapes drive transcriptomic alterations and so, they determine the basal and classical phenotypes. In the classical subtype several TFs including GATA6, FOS, FOXP1, and FOXP4 act at the super-enhancer level to regulate gene expression, they also regulate the expression of other TFs (HFFs, PDX1, MNX1, PPARs) with functions in pancreatic morphogenesis and lipid metabolism. In the basal subtype, which has a worse prognosis, MET was found to regulate basal-specific super-enhancers, gene networks downstream of MET include TFs such as MYC, MYBL1, E2F1, and SNAI2 known to play a role in proliferation and the epithelial to mesenchymal transition. As the nature of PDAC subtypes is not genetic, it could be possible to interconvert both subtypes just by inactivation of MET in basal or GATA6 in classical samples.

As described above, the origin of PDAC subtypes cannot be explained by the common genetic aberrations observed in this pathology. Nicolle et al. (5) demonstrated using a multi-omic approach (RNA-Seq, miRNA-Seq, Exome-Seq, methylation analysis, and SNP chips) in patient-derived xenografts, that both basal and classical PDAC subtypes could be classified by specific alterations in their DNA methylation patterns. These results indicate that the main phenotypic outcomes in PDAC are epigenetically rather than genetically established. For example, key players in PDAC heterogeneity are the super-enhancers (7). These are clusters of enhancers located in the same region of the genome that mediates cell identity and function (42). The nucleosome remodeling SWI/SNF complex can regulate these super-enhancers and interestingly, genetic alterations in members of this complex are frequent among PDAC tumors. When the SWI/SNF complex is unable to assemble correctly it cannot oppose the polycomb repressive complex localized to the promoters and typical enhancers of differentiation genes. However, residual functional SWI/SNF complexes remain as super-enhancers of genes involved in the maintenance of cell identity, and this disequilibrium promotes tumorigenesis (43). In concordance with Nicolle et al. (5), Lomberk et al. (7) identified that in the classical subtype of PDAC TFs such as GATA6, FOS, FOXP1, FOXP4, KLF4, ELF3, NFIX, CUX1, and SSBP3 seem to be implicated in the upstream transcriptional regulation of other TFs with functions in pancreatic morphogenesis and lipid metabolism pathways, but were also found to regulate gene expression at the super-enhancer level (Figure 1). GATA6 has a proposed oncogenic function, as its overexpression was found as a consequence of frequent genomic copy number gain in some pancreatic cancer cells (44). However, it was recently reported that in fact, GATA6 inhibits de-differentiation and the epithelial to mesenchymal transition, while GATA6 silencing increases the metastatic capacity of PDAC cells by regulating different TFs including FOXA1/2 (45). Moreover, loss of GATA6 in primary PDAC samples was linked to shorter overall patient survival. On the other hand, in the basal phenotype the hepatocyte growth factor receptor (MET) acts to upregulate super-enhancers. In the networks located downstream of MET there are several TFs involved in proliferation (MYC, MYBL1 and E2F1) and in the epithelial to mesenchymal transition (SNAI2) which could play a key role in the development of basal tumors.

### The Epigenetic Landscape Drives Distinctive Gene Expression Profiles

Remarkably, aberrant DNA methylation patterns and/or deregulation of miRNA epigenetic modifications correlate with alterations in subtype-specific pathways, further supporting the hypothesis that epigenetics drives the PDAC phenotypes (5, 46). An integrative analysis performed by Nicolle et al. (5) concerning gene expression and genome methylation data from different studies (5, 47–50), showed a clear parallelism between the methylome and transcriptome. On one hand, cells from basal tumors are characterized by high expression levels of genes related to cell cycle and glycolysis pathways, and both tumoral and stromal cells have an overrepresentation of pathways involved in the interaction with nearby tissues. Moreover, in both tumoral and stromal cells from basal samples the WNT signaling pathway is also highly deregulated by overexpression of its ligands and downregulation of inhibitors. On the other hand, pathways upregulated in the classical phenotype are also active in normal pancreatic cells or other types of gastrointestinal cells. Thus, characterization of the epigenetic landscape and gene expression is a powerful tool not only as a marker of clinical evolution, but also to understand the functional mechanisms in tumoral cells and find new therapeutic targets.

These observations are in agreement with several previous studies which have successfully classified PDAC tumors into specific subtypes based on tumoral gene expression profiles, indicating that unlike genetic aberrations, the transcriptome as a reflection of the methylome, correlates to therapeutic response and clinical outcome. Collisson et al. (47) described three intrinsic subtypes: classical, quasi-mesenchymal, and exocrine-like. More recent studies with a larger sample size have extended the subtypes already described. The classification described by Moffitt et al. (48), that used only PDAC samples, was able to classify tumors independently of sample purity (46) into two subtypes with prognostic and biological relevance: classical-like and basal-like. Another stratification by Bailey et al. (49) proposed four subtypes: squamous, immunogenic, pancreatic progenitor, or aberrantly differentiated exocrine. Another tumoral classifier expression signature which correlates with clinical outcome was proposed based on the expression of MYC gene: MYC-low and MYC-high subtypes with higher and lower overall survival, respectively (17). The MYC-high subtype has higher levels of proliferation, a lower state of differentiation and shorter survival time compared to the MYC-low subtype. The basal/classical stratification of Moffitt et al. (48) has gained consensus in the last few years, as it has been validated by several independent cohorts studies.

Recent data from multi-omic approaches (5, 7) highlight that epigenetic modifications play a key role not only in carcinogenesis but also in PDAC heterogeneity. Thus, the evolution of PDAC could be thought of as a phenomenon that begins with several alterations at the genetic level, which combined with factors from the environment and tumor microenvironment defines an epigenetic landscape outlining the PDAC phenotypes. Characterization of both the epigenetic landscape and its resulting transcriptome allow the classification of PDAC samples into the less aggressive classical subtype or the more aggressive basal subtype. Therefore, the different chromatin states could serve as potential phenotypic, diagnostic, and prognostic markers; and moreover they could be a useful tool to identify new therapeutic targets.

### Possible Sources of Samples for the Identification of Epigenetic Biomarkers of Response to Chemotherapy

A great advantage of stratifying patients according to epigenetic marks instead of RNA expression is that DNA is much more stable. It is known that solid tumors release cell-free DNA into the circulation and its DNA methylation pattern mirrors the tumoral profile. Aberrant circulating methylated DNA has already been studied for most types of cancers because of its clinical implications: it can be analyzed directly from plasma or serum samples without the need for any surgical or invasive procedures (51). In addition, as DNA methylation is often an early event in carcinogenesis, methylated DNA could be a sensitive marker for the early stage of disease (52). Several studies have focused on utilizing this strategy for the early detection of PDAC (53–55). With the same objective, other epigenetic modifications have also been studied in PDAC blood-based samples, for example histone modifications, by analyzing circulating nucleosomes (56) and miRNAs (57). These non-invasive techniques could be used to molecularly characterize subtype-specific epigenetic signatures, leading to early stratification and treatment selection. However, further studies should be performed to test their efficacy.

Immunodeficient mice xenografts established from patient biopsies can be useful for the molecular characterization of tumor samples; however, graft growth can take many months which is not a clinically compatible timeframe. An alternative method to the xenograft is the culture of organoids, and in the last few years this has become a helpful technique for translational medicine in cancer research. This 3D system recapitulates different aspects of tumoral cells such as tissue architecture, cell-cell interactions, polarity, and cellular heterogeneity by conserving the mutational profile, while 2D models do not. Another advantage is that they can be produced not only from resectable PDAC macro-biopsies but also from fine-needle aspiration (FNA) micro-biopsies (58). Moreover, organoid culture could be performed in a clinically compatible timeframe, as their preparation directly from PDAC biopsies could take only 2 to 3 weeks (59). The pure material that is obtained after growth in selective media can be used for subsequent molecular characterization, including transcriptomics and epigenomics. Moreover, to generate a complete 3D model of the tumoral environment, pancreatic cancer organoids can be co-cultivated with stromal fibroblasts and/or immune system cells (cancer-associated fibroblasts and T-cells) (60). As this approach takes into account the whole complexity of the tumor it is very useful for accurate drug testing.

A recent study found that analysis of the gene expression profile of PDAC organoids is a powerful tool for molecular pharmacotyping (61). Gene expression signatures for sensitivity to the current drugs used in PDAC treatment were defined, especially to gemcitabine, oxaliplatin, and paclitaxel, as adjuvant therapies and to treat advanced disease. Tumors resistant to one drug could be sensitive to another; therefore these signatures may allow the development of a more adapted treatment for each patient. Although oxaliplatin resistance was more common in the basal subtype, pharmacotyping signatures did not overlap with the phenotypic signatures, suggesting that different pathways are involved in clinical outcome and response to therapy. This pioneering study showed that using organoids to predict the response to chemotherapy is a feasible approach and, most importantly that it is possible to identify molecular signatures which allow a rapid evaluation of phenotypic markers that can then be utilized to select the best chemotherapy based on patient stratification. These results encourage more studies, not only at the transcriptomic but also at the epigenomic level, to search for biomarkers of response to chemotherapy.

## Epigenetics as a Tool to Search for New Therapeutic Targets

Understanding heterogeneity in PDAC and the epigenetic mechanisms underlying it, brings with it the possibility to search for new potential therapies with more specific targets based on tumor phenotype. However, due to their particular microenvironment, pancreatic tumor cells adapted to survive under intense metabolic pressure (62). The extensive PDAC stroma exhibits low vascularization conducive to hypoxia and nutrient deprivation with subtype-specific characteristics. In the classical subtype, small molecula transporters such as the glutamine SLC1A1 transporter and cholesterol transporters like NPC1L1, are over-expressed as a consequence of DNA hypomethylation. Nicolle et al. (5) proposed an interesting approach: to target the highly epigenetically deregulated NPC1L1 using its well-known inhibitor ezetimibe, a drug that is safely and effectively used to treat hypercholesterolemia (63). Interestingly, no previous studies suggest a role for this cholesterol transporter in pancreatic cancer, thus NPC1L1 emerges as a novel target from epigenetic characterization. However, cholesterol metabolism is already known to play an important role in PDAC. It has been reported that a high intake of cholesterol might increase the risk of pancreatic cancer (64). Moreover, cholesterol uptake is a key metabolic pathway for the maintenance of tumoral cholesterol distribution, which is essential for PDAC progression (65). The results obtained by Nicolle et al. (5) in organoids and xenografts after treatment with ezetimibe indicated the efficacy of this drug alone and in combination with gemcitabine. As ezetimibe is a cholesterol competitor, classical tumors that express higher levels of NPC1L1 showed lower sensitivity compared to basal tumors, suggesting different subtype responses. This is a proof of concept that using epigenetic characterization to identify pathways that are aberrantly regulated, especially those that are indispensable for cell survival, is a good strategy to find target candidates against PDAC. Other epigenetically deregulated pathways are also potentially druggable. For example, the DNA methylation of several effectors and inhibitors from the WNT signaling network is known to be altered in the basal subtype of PDAC. In basal tumors, inhibition of the WNT pathway is promising, as several therapeutics targeting WNT signaling are in preclinical phases or clinical trials for the treatment of cancers associated with WNT alterations (e.g., vantictumab, cirmtuzumab, and rosmantuzumab) (66).

In addition, new strategies could focus not only on the difficult mission of eliminating all cancer cells, but also on converting the phenotypes with the worst outcomes to another phenotype with a better prognosis. With this aim Lomberk et al. (7) inactivated MET using small interfering RNA (siRNA) in tumors classified as the basal subtype. The authors showed that this inactivation had an effect at the super-enhancer level of regulation and consequently caused a progression from an aggressive to a more benign tumor. The strength of this strategy is that MET is an intermediate between the epigenome and transcriptome so, the target is not a specific pathway but neither a non-specific modification of the epigenetic marks of the whole genome. Interestingly, in other cancers such as non-small cell lung cancer and hepatocellular carcinoma, anti-MET therapy with monoclonal antibodies, and small-molecule inhibitors has already been used in clinical trials with a significant efficacy and low toxicity profile (67). This study encourages the development of future therapies targeting the MET pathway, alone or in combination with standard therapies (Figure 1). Finally, these results are the first evidence that it is possible to modify the tumor phenotype; thus, we can hypothesize that it is also possible to alter the pharmacotype and turn a resistant cell into a sensitive one.

## Conclusions and Perspectives

One of the most important problems facing the treatment of PDAC is its great heterogeneity. This heterogeneity has an epigenetic origin; it is not the result of genetic aberrations as had previously been thought. We can continue trying to develop new and more efficient drugs that save all PDAC patients, but this is a utopia. Instead, according to the tumor phenotype we can select the most suitable treatment for each patient or group of patients, using drugs already available. Epigenetic characterization of the tumor using circulating DNA or primary culture organoids could allow us to predict clinical outcome and select a therapeutic strategy: current anticancer drugs or existing drugs used in other pathologies, alone or in combination, could be used to eliminate the tumor, convert it to a less aggressive phenotype or increase its sensitivity to chemotherapy.

## Author Contributions

NJ, JI, and ND conceived, wrote the manuscript, and create the figure.

### Conflict of Interest Statement

The authors declare that the research was conducted in the absence of any commercial or financial relationships that could be construed as a potential conflict of interest.
